# Pharmacogenomic (PGx) Counseling: Exploring Participant Questions about PGx Test Results

**DOI:** 10.3390/jpm10020029

**Published:** 2020-04-23

**Authors:** Tara Schmidlen, Amy C. Sturm, Laura B. Scheinfeldt

**Affiliations:** 1 Coriell Institute for Medical Research, Camden, NJ 08003, USA; 2 Genomic Medicine Institute, Geisinger, Danville, PA 17822, USA; asturm@geisinger.edu

**Keywords:** pharmacogenomics, return of results, genetic counseling, qualitative

## Abstract

As pharmacogenomic (PGx) use in healthcare increases, a better understanding of patient needs will be necessary to guide PGx result delivery. The Coriell Personalized Medicine Collaborative (CPMC) is a prospective study investigating the utility of personalized medicine. Participants received online genetic risk reports for 27 potentially actionable complex diseases and 7 drug–gene pairs and could request free, telephone-based genetic counseling (GC). To explore the needs of individuals receiving PGx results, we conducted a retrospective qualitative review of inquiries from CPMC participants who requested counseling from March 2009 to February 2017. Eighty out of 690 (12%) total GC inquiries were focused on the discussion of PGx results, and six salient themes emerged: “general help”, “issues with drugs”, “relevant disease experience”, “what do I do now?”, “sharing results”, and “other drugs”. The number of reported medications with a corresponding PGx result and participant engagement were significantly associated with PGx GC requests (*p* < 0.01 and *p* < 0.02, respectively). Our work illustrates a range of questions raised by study participants receiving PGx test results, most of which were addressed by a genetic counselor with few requiring referrals to prescribing providers or pharmacists. These results further support a role for genetic counselors in the team-based approach to optimal PGx result delivery.

## 1. Introduction

Pharmacogenomics (PGxs) is a rapidly growing segment of precision medicine projected to reach a market size of USD 9.9 billion globally by 2025 (https://www.researchandmarkets.com/reports/4801556/global-pharmacogenomics-market-2019-2025). There are over 250 drugs approved by the United States Food and Drug Administration (FDA), with labels containing pharmacogenetic information (https://www.fda.gov/drugs/science-and-research-drugs/table-pharmacogenomic-biomarkers-drug-labeling; accessed on 22 January 2020). In October 2018, the FDA granted 23andMe the first and only authorization to sell a PGx test that reports on 33 pharmacogenetic variants directly to consumers [1]. Aside from 23andMe, a growing number of clinical laboratories currently offer PGx testing, with many allowing consumers to initiate testing via an independent ordering provider rather than their personal physician. Outside of the consumer-driven genetic testing marketplace, there are large research initiatives like the National Institutes of Health’s *All of Us* Research Program that is planning to return pharmacogenomic test results to as many as one million participants (https://news.nnlm.gov/psr-latitudes/nih-all-of-us-research-program-plans-genome-sequencing-and-genetic-counseling-for-participants/; posted on 21 August 2019). As the availability and use of pharmacogenomic (PGx) information in healthcare increases, a better understanding of the informational needs of individuals receiving this information will be necessary to help guide PGx result delivery.

There is relatively little information published on the extent to which patients understand PGx test results. Lemke and colleagues [2] surveyed 57 patients from NorthShore University Health System in suburban Chicago, Illinois, USA, who had undergone a 19-gene panel PGx test through either a PGx clinic or by direct access in-home testing. Participants were mostly female (72%), Caucasian (98%), and educated (93% with some college or more). Clinic patients received a discussion of benefits, risks, and limitations of PGx testing from a PGx specialist prior to sample collection in the clinic. Direct access patients watched a 4 min video covering the same topics prior to sample collection in their home. Results were returned to patients and placed in the electronic health record with the PGx clinic team available to answer questions. When surveyed 4 to 8 weeks after result receipt, the majority (63%) of these patients reported that they strongly agreed or somewhat agreed with the statement “I have a clear understanding of my test results from PGx testing”. About 25% markedly disagree or strongly disagreed in response to this statement. Forty percent reported feeling confused by their PGx test results “often” or “sometimes”. Forty percent indicated that they “looked up additional information” about their PGx results after discussing them with a provider, and 36% said that they wanted more follow-up discussion on their PGx results with their healthcare provider. The only difference in findings between the clinic and direct access was that clinic patients had a higher self-perceived understanding of their PGx test results than direct access patients (77.3 vs. 51.5%; *p* = 0.06). A third of patients commented in the open text at the end of the survey, and the need for additional education in results explanation was a key issue raised.

Haga and Liu [3] conducted an online survey of 99 individuals who had subscribed to a newsletter offered by Genelex, a United States-based commercial PGx laboratory. Respondents were mostly female (76%), Caucasian (93%), educated (60% with Bachelor’s degree or higher), and older (majority 50–59 years, range 18–80+). Most (91%) had PGx testing through Genelex, and 48% had tested more than one year from survey completion. Most (87%) had received their results in-person with the remainder by phone or by email. Approximately half of the respondents reported viewing one or more of four Genelex educational web pages. Forty-three percent felt that they understood their PGx test results very well, 39% indicated that they “somewhat” understood their PGx results, and 11% reported that they did not really understand their PGx results. No significant association between education level or educational web page viewing and understanding of PGx test results was observed.

The Mayo Clinic Right Drug, Right Dose, Right Time Protocol (RIGHT Protocol) is a preemptive PGx study of 1013 individuals selected from the Mayo Clinic Biobank based on age, sex, and race. Olson et al. [4] surveyed 869 participants who had received *CYP2D6* metabolizer statuses via the RIGHT Protocol. Respondents were mostly Caucasian (98%), female (55%), educated (57%—4 or more years post-secondary), and aged 58.9 ± 5.5 years on average. Study participants received a result summary letter via mail along with an educational brochure with one page of information on how to sign in to the Mayo Clinic Patient Portal to access results, two pages of education on PGx testing and *CYP2D6*, a 9-page survey and a postage-paid envelope to return the survey. The majority (67%) of study participants responded that they either completely or mostly understood their *CYP2D6* result when asked, “How well do you feel you understand or don’t understand your *CYP2D6* test result?” About a third (33%) responded that they either only somewhat understood (26%) or did not understand their result at all (7%). Education was the only relevant predictor, with those reporting high school or less or some college being 1.6 times as likely to report understanding somewhat or not at all compared with those who had a four-year college degree or greater education level. Over half (53%) of the 499 participants who logged in to view their result on the patient portal agreed with the statement, “It was easy to understand my pharmacogenomic result in the Patient Portal”, while 33% disagreed. When asked to comment on confidence level in their ability to explain their *CYP2D6* result to a friend or family member, 38% responded “somewhat confident”. Responses varied by education level with 13% of high school or less and 23% graduate/professional degree holders reporting that they were “extremely confident”, while 30% of high school or less and 18% of graduate/professional degree holders reported being “not at all confident” in their ability to explain results to others.

Limited studies to date have commented on patient understanding of PGx test results. Several have found that even among well-educated patients, there remain significant gaps, and, therefore, opportunities for improvement upon PGx result return approaches. Here we describe the informational needs of individuals receiving PGx test results through an online web portal as part of their participation in the Coriell Personalized Medicine Collaborative (CPMC), a large prospective precision medicine study.

## 2. Materials and Methods

We conducted a retrospective qualitative review of genetic counseling session summaries from participants who requested counseling to discuss their CPMC PGx results. The Coriell Institute for Medical Research Institutional Review Board reviewed and approved this study. As a retrospective review of existing CPMC participant records, no additional participant consent was required.

The CPMC, described in detail in Keller et al. [5], is a prospective research study that assesses the impact of personalized genetic information on disease risk and medication management [6] on health behaviors and outcomes. CPMC participants must be at least 18 years of age, have a valid, personal email address, and attend or view a 45 min long informed consent PowerPoint presentation. The informed consent presentation provides an explanation of personalized medicine, study design and participation requirements, risks and benefits, examples of potentially actionable health conditions and drug-gene pairs reported to participants (e.g., coronary artery disease; CYP2C19, and Plavix), and examples of excluded health conditions (rare, single-gene Mendelian diseases and conditions with no available medical or behavioral risk reduction). The CPMC defines a “potentially actionable” condition as a condition for which the risk is likely to be mitigated by either behavior or lifestyle modifications (diet and exercise, smoking cessation) or by medical actions such as changing a drug or drug dose, increased screening, preventative treatment, or early intervention [5,6].

**Figure 1:**
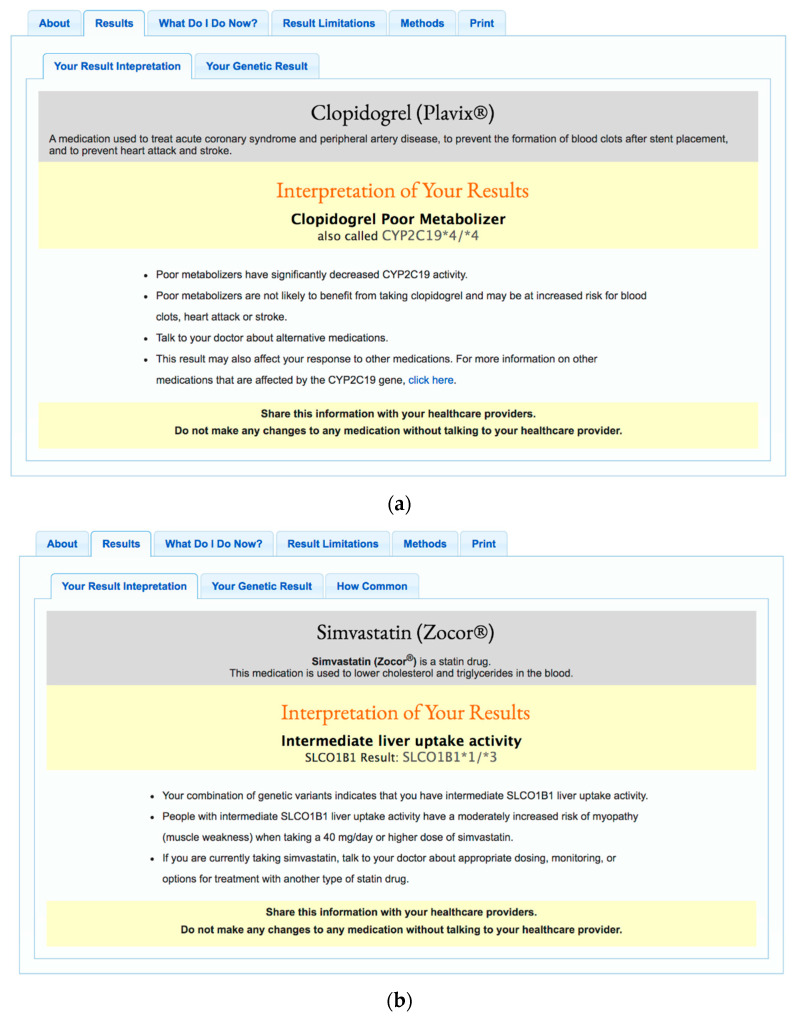
Example Coriell Personalized Medicine Collaborative (CPMC) pharmacogenomic (PGx) reports. Figure 1 displays example PGx reports for (**a**) clopidogrel and (**b**) simvastatin.

Participants provide a saliva sample for genomic analysis (Affymetrix™ Genome-Wide Human SNP Array 6.0 and DMET Plus Array genotyping chips). The DMET Plus Array assays over 1900 genetic markers located in genes involved in drug absorption, distribution, metabolism, and elimination (ADME) [7]. Participants also complete mandatory online questionnaires about their medical history, family history, medication use, demographics, and lifestyle. Those who complete all required questionnaires are invited to view their results through a secure web-based portal. During the 6-year time frame captured by this study, participants received the following PGx results: warfarin (CYP2C9, VKORC1, CYP4F2), clopidogrel (CYP2C19), proton pump inhibitors (CYP2C19), thiopurines (TPMT), simvastatin (SLCO1B1), metformin (ATM), and celecoxib (CYP2C9). Example reports for clopidogrel and simvastatin are displayed in Figure 1.

### 2.1. Participants

The CPMC is comprised of several cohorts [5,8,9,10,11,12,13]: a CPMC community cohort recruited from the general population, a cancer (breast and prostate) cohort recruited through oncologists at Fox Chase Cancer Center, a chronic disease (congestive heart failure and hypertension) cohort recruited through primary care physicians or cardiologists at Ohio State University Medical Center, a community cohort recruited through Ohio State University, and a cohort recruited through the United States Air Force. All participants are at least 18 years of age and have given written, informed consent to enroll in the study. No participants were excluded based on comorbidities. In total, information from 690 participant requests for genetic counseling support was included in the current analysis.

### 2.2. Procedures

Participants included in the current analysis received email invitations to view online genetic risk reports for up to 27 actionable complex diseases and 7 drug-gene pairs. Participants could elect to view or not view each report and were encouraged, but not required, to share and discuss their results with a healthcare provider. Participants had the option to request a telephone genetic counseling session paid for by the study via email, by phone, or through a secure web portal. All written requests for counseling (email or web portal requests) and subsequent written communications were stored verbatim, while the content of telephone genetic counseling sessions was captured in the form of detailed session summaries which identified participant questions, issues, and information provided. Counseling notes for 30 telephone genetic counseling sessions and email transcripts for 50 email inquiries made by CPMC participants between April 2009 and February 2017 were included in this analysis. All genetic counseling sessions included in this study were conducted by two licensed board-certified genetic counselors employed by the Coriell Institute for Medical Research.

### 2.3. Data Analysis

Genetic counseling session summaries were coded and analyzed via a general inductive approach to identify themes related to the research questions and study aims. Study investigators compared coded genetic counseling session summaries, modified codes as needed, and developed rules and definitions to ensure coding consistency. Codes from this finalized codebook were then applied to each of the 80 genetic counseling session summaries by two study investigators (TS, AS) with an inter-coder reliability of 91%. Line-by-line codes were compared for all session summaries, and all instances of disagreement were resolved by consensus.

We used logistic regression to model whether a participant was more likely to request PGx genetic counseling as the outcome, with recruitment cohort, gender, education, income, occupation, and age as demographic covariates, and number of medications, number of medications with CPMC risk reports, and number of viewed CPMC risk reports as independent variables. We used the step function in R to choose the demographic model with the lowest AIC: request_PGx_GC~ age + cohort, and then tested each independent variable with this model.

## 3. Results

**Figure 2:**
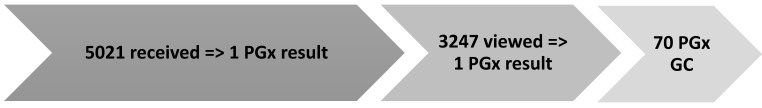
Participation in PGx genetic counseling.

We conducted a retrospective qualitative review of genetic counseling session summaries from participants who requested counseling to discuss their CPMC PGx results. As of 30 September 2015, 5021 had completed the required baseline surveys, had their sample genotyped, and were provided with at least 1 PGx result report. Of those 5021 participants with available PGx results, 4779 participants (95%) chose to view at least one risk report, and 3247 participants (65%) chose to view at least one PGx result. Of those 4779 who chose to view at least one report, 569 (12%) participants submitted at least 1 request for genetic counseling to a CPMC genetic counselor. Of those 3247 who chose to view at least one PGx result, 70 participants (2%; also see Figure 2) submitted at least 1 request for genetic counseling to a CPMC genetic counselor (62 participants submitted 1 request, 7 participants submitted 2 requests and 1 participant submitted 4 requests).

Overall, 73% of the 5021 participants with available PGx results were taking at least 1 medication. On average, these participants were taking 3 medications. A smaller subset, 22% of participants, were taking at least 1 of the 7 drugs reported on in the study. A comparable proportion of the participants requesting genetic counseling for a PGx result were taking at least 1 medication (52/70; 74%). Compared to the 5021 participant pool, a higher proportion of participants requesting genetic counseling for a PGx result were taking at least 1 of the 7 drugs reported on in the study (30%; 21/70).

The 5021 participants who received at least one PGx result were primarily Caucasian (88%), middle-aged (mean: 47, range: 18–94), females (57%) with a Bachelor’s degree or higher (69%); 35% were employed in a health or science occupation, and 18% reported a household income greater than USD 100,000 per year. Additional demographic characteristics of the 5021 CPMC participants and the subset of participants that requested genetic counseling are provided in Table 1 and Table 2, respectively.

**Figure 3:**
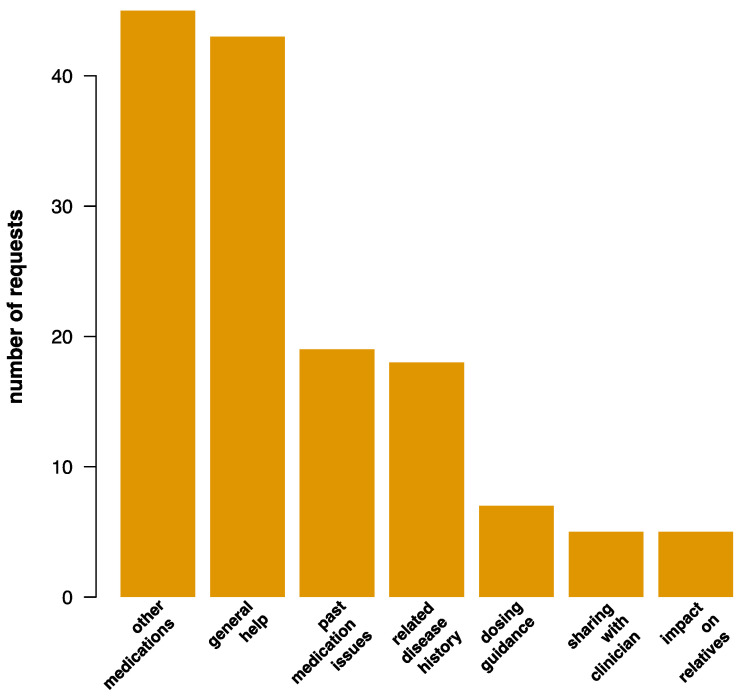
Broad PGx GC topical categories.

Eighty out of 690 (12%) total GC inquiries were focused on the discussion of PGx results. Qualitative analysis of these 80 GC session summaries revealed six main themes: (1) general help (2) issues with drugs (3) relevant disease experience (4) what do I do now? (5) sharing results, and (6) other drugs. Forty-three (54%) participants had general questions about their PGx results, while only 7 (9%) were looking for specific guidance on dosing or drug selection. Past issues with drug side effects (*n* = 14, 18%), dosing (*n* = 2, 3%), and efficacy (*n* = 3, 4%) were also mentioned; some alluded to a personal (*n* = 12, 15%) or family history (*n* = 6, 8%) of diseases treated by drugs reported on in the CPMC study. Seventeen participants (21%) were interested in the availability of PGx results for other non-study related drugs, while 28 (35%) asked about the impact of currently available PGx study results on other drugs. Some indicated sharing their PGx results with a doctor (*n* = 5, 6%), and some questioned the impact of their PGx results on family members (*n* = 5, 6%) (Figure 3).

For the 569 unique participants that requested genetic counseling, recruitment cohort and age were the only demographic factors retained for the regression modeling (see Methods). The total number of CPMC risk reports viewed by a participant was marginally associated (*p* = 0.02) with a PGx counseling request, and the number of reported medications with a corresponding CPMC PGx risk report was significantly associated with PGx GC requests (*p* = 0.007) (Table 3). The following quotes illustrate key concepts and summaries of participant remarks representing each theme.

### 3.1. General Help

The majority of participants requesting genetic counseling for a PGx result (54%) were seeking general assistance with understanding their CPMC PGx result reports. Participants often alluded to uncertainty regarding whether they should or should not be taking a given medication based on their study result.

“I’m a little confused about the warfarin medication results. Currently I do not take warfarin. So, under (Your Result Interpretation) it states “Intermediate Dose of Warfarin MAY be Needed Based on Your Combined Genetic Result: CYP2C9*1/*1, VKORC1-AG, CYP4F2-GG”. Is this advising me to start taking warfarin and seek a prescription...or simply like the other reports keep this in mind if the need arises that I need to start this drug?”

Some were confused by an “uncertain metabolizer” result, which was issued in instances of rare alleles, uncertain phase, or missing single nucleotide polymorphism (SNP) data on research-grade testing.

“In this test my result was referred to as “CYP2C19 *Uncertain (Clopidogrel Metabolizer Status Uncertain)” While I understand that my genetic combination is not known, that was stated directly in the results, I am just curious as to what about it is unknown. Does it not have a common singular response, or is this combination (7 out of 100 according to the “How Common” part of the result) not studied enough?”

Others were interested in gaining a better understanding of the details or terminology provided within their report:

“It is unclear what AA, GG, TT, etc. mean.”

*“You use the term -Clopidogrel Extensive Metabolizer- which offhand is meaningless to me, but after looking up the term on the web, it apparently means that if I took it, my body would use it well.”*

### 3.2. Issues with Drugs

A quarter of participants requesting genetic counseling for a PGx result did so because of a personal history of experiencing side effects of a drug (*n* = 14, 18%), dosing issues with a prescribed medication (*n* = 2, 3%), or of lack of efficacy of a prescribed medication (*n* = 3, 4%).

*“I have high cholesterol and have had adverse effects with other statins. I have not used Simvastatin and would like to discuss if my results recommend trying this drug.”*

*“I’ve been taking Nexium for weeks and it doesn’t seem to be helping my GERD.”*

### 3.3. Relevant Disease Experience

About a quarter of participants (23%) referenced either a personal or family history of a disease being managed by one of the study drugs within their PGx genetic counseling inquiry.

*“I am interested in more information regarding my warfarin results. I’m a physician and I’m fascinated by the results. During my residency, I suffered a significant superficial thrombosis is some large varicose veins. Due to the amount of venous dilation, I was treated with warfarin. If my memory serves me correctly, I required 10 days of lovenox therapy because of the difficulty getting my INR to a therapeutic level; I believe my final dose was 20 mg daily.”*

*“I have a family history of stroke (mother) and she is currently on this medication. In the future, if this drug is offered, should I decline as it does not look effective in my case?”*

### 3.4. What Do I Do Now?

Nine percent of PGx genetic counseling requests were seeking guidance on more concrete next steps that should be taken in light of the results. These included determining whether a dose change may be needed for a currently prescribed medication or whether a change in treatment approach to a different drug may be indicated by the results. These individuals were referred back to their prescribing healthcare provider or pharmacist for medication management guidance.

*“I am taking Warfarin and would like to know what is a safe dosage for me and what range I should be in for protection against blood clots.”*

*“My husband had a stroke and has been told to take Plavix. He is a ultra-rapid metabolizer. What should we do?”*

### 3.5. Sharing Results

A few participants (*n* = 10, 12%) referenced a plan to share their PGx results, either with family members (*n* = 5, 6%) or with their doctor (*n* = 5, 6%). In some cases, participants were seeking guidance on which PGx results were worth sharing with family members or physicians. A few participants mentioned having already shared a PGx result with a physician who also did not know what to do with the information.

*“I need info on my CYP2C9 results and how they are genetically carried to my children. I have a child in a critical care that may need this info.”*

*“As an intermediate metabolizer of Plavix, I understand the implications of this finding if my medical care called for treatment in the future and I will inform my physician about this finding. I am less certain if the CPMC thinks it wise that I also talk to my physician about my current use of omeprazole for mild GERD for which, If I understand the findings correctly, I may currently be under-dosed.”*

*“I took this result (Simvastatin) to my PCP and he did not know what to do with all this info, and I did not know either.”*

### 3.6. Other Drugs

Greater than half of all PGx result inquiries alluded to an interest in receiving additional PGx information. This included requests for future results on drugs not currently included in the CPMC study (*n* = 17, 21%) or interest in whether their PGx results impacted the metabolism of drugs that were not specifically referenced on their study result report (*n* = 28, 35%). CPMC genetic counselors responded to requests for additional PGx information by indicating whether the drug had been considered or approved by the study advisory board for eventual return to participants. CPMC genetic counselors did not attempt to independently interpret PGx results for drugs not included in the report interpretation but rather reminded participants of the report disclaimer stating that their result interpretation applied only to the drug(s) listed on the study report. Among the most common requests for future results were psychiatric medications (5/17, 29%). Additional medication types requested included antibiotics, bone health medications (Prolia, Xgeva), benzodiazepines (diazepam, valium), blood pressure medications (lisinopril), chemotherapeutic agents (Fulvestrant), pain medications (NSAIDs, Vioxx, codeine), insulin, other cholesterol lowering drugs (Lipitor, Zetia), prednisone, and tamoxifen. The impact of CPMC PGx results on other drugs was most commonly related to whether their SLCO1B1–simvastatin results also applied to other statin drugs. Others were interested in whether their CYP2C19–clopidogrel results also influenced the metabolism of other blood-thinning medications like aspirin.

*“I was wondering if you are doing any work in the area of brain chemistry. I have been diagnosed with severe depression and we are searching for the optimal medication.”*

*“I was wondering if the study will be doing tests for which antibiotics might not work with my genes?”*

*“Based on my reports I should not take Zocor (Simvastatin). Any information about Lipitor before I start taking it?”*

## 4. Discussion

Through a qualitative exploration of genetic counseling interactions with participants in a large precision medicine biobank study, this study identified common questions that individuals have when receiving preemptive PGx test results. Most participants (54%) had general questions about how to interpret their PGx results, while only 9% were looking for specific guidance on dosing or drug selection that required referral to a healthcare provider or pharmacist. Other inquiries were related to past issues with drug side effects, dosing and efficacy, personal or family history of diseases treated by drugs reported on in the study, impact of study results on family members, and sharing results with healthcare providers. Several participants expressed interest in receiving additional PGx results for other drugs not included in the study. These results highlight for genetic counselors and other health care providers gaps in understanding of PGx test results, participant reaction to PGx results, as well as the desire that many expressed for additional PGx result information beyond what was offered through this study.

Consistent with existing literature on patient understanding of PGx test results, this study identified gaps in understanding of PGx test results even among a mostly highly educated study population [2,3,4,14]. Most participants requesting genetic counseling (54%) were seeking general assistance with understanding their PGx result reports, often alluding to uncertainty regarding whether they should or should not take a given medication in the future based on their results. The expectation for PGx test results to have the potential to inform physician prescribing decisions in a way that maximizes drug efficacy while limiting adverse reactions has been captured in several other studies, including studies of participants who have received PGx results [2,3,14,15,16,17].

Uncertain results, which were issued in instances of rare alleles, uncertain phase, or missing genetic data, were a source of confusion as was some of the terminology used on reports to describe metabolizer status. Others have also documented confusion with PGx test results and the metabolizer status terminology present in the interpretation of those PGx test results. Lee et al. [16] asked focus group participants with either prior PGx exposure or none to review educational handouts for clopidogrel and simvastatin PGx results. Participants expressed concern that only four categories were examined (poor metabolizer, intermediate metabolizer, extensive/normal metabolizer, ultra-rapid metabolizer) and wondered if that meant that the testing was either incomplete or too limited in scope to be useful. The Mayo Clinic Right Drug, Right Dose, Right Time (RIGHT) Protocol study surveyed 869 participants on their understanding of their *CYP2D6* PGx test results and accompanying educational materials [4]. They asked participants what would have made their results more helpful, and the most common suggestion was to use layperson’s terms (e.g., extensive metabolizer is not as clear as “normal” metabolizer), followed by personalizing the result report (e.g., list the drugs that are impacted by the result) and simplifying the layout and content of the results report (e.g., add a graph showing where the result is in relation to a “normal” result). Current nomenclature that has replaced “extensive metabolizer” with “normal metabolizer” may reduce confusion.

As was expected, participants with either a personal or family history of a disease managed by one of the study drugs were among those placing a PGx genetic counseling request. Many participants requesting genetic counseling for a PGx result had a personal history of drug side effects, dosing issues, or lack of response to a medication and were curious if their PGx results validated or explained that experience.

About 70% of participants in a study that surveyed patients who had PGx testing in either a PGx clinic or via a direct access in-home test reported feeling validated about their history of previous drug side effects or lack of efficacy following receipt of PGx test results [2]. Participants in a focus group study on patient, physician, and pharmacist opinions on PGx conducted by Frigon et al. [15] commented on the potential for PGx test results to limit the experience of having a physician discount a patient’s report of a drug side-effect as a “psychosomatic” event rather than a real drug side effect. The notion of PGx results potentially leading people to conclude that any and all adverse events from a drug was due to their genetic make-up was noted by Lee et al. [16] in their focus group study comparing the attitudes and perceptions of individuals exposed to PGx-guided care versus those of individuals with traditional care. On the other hand, some studies have found that individuals receiving PGx test results may be more medication compliant and willing to tolerate some side effects if PGx testing was utilized to assist with drug and dose selection [2,16,18,19]. Results from a study by Haga et al. [14] lend further support to the hypothesis that PGx testing may improve patient adherence to medications. They studied participants who had pharmacist-assisted PGx testing and completed pre- and post-test surveys on their experiences and beliefs about prescription medicines and perceived risk and benefits of PGx testing. More than half of these participants post-test reported feeling confident that medications they were prescribed going forward would be safe and would improve their health condition compared with past prescriptions issued prior to their PGx testing. Similarly, Lemke et al. [2] found 73% of patients reporting greater confidence in medication efficacy and safety following PGx testing compared with prescriptions issued prior to testing.

Personal history of a drug side effect has been noted as a driver of PGx interest among individuals without prior PGx test exposure as well. In a 2012 telephone survey study looking at attitudes toward PGx testing in the United States [17], those who had experienced a side effect from a prescribed drug were more likely to have a strong interest in PGx testing, even after potential risks of PGx testing (e.g., privacy, confidentiality, blood draw) were reviewed.

More than half of all PGx genetic counseling inquiries mentioned an interest in receiving additional PGx information. These included requests for future results on drugs not yet included in the study (psychiatric medications), as well as interest in whether their PGx results impacted other drugs that were not specifically referenced on their result report (other statins, or other blood thinners). Other studies have also demonstrated that participants with exposure to PGx results have a strong receptiveness toward the use of pharmacogenomics and desire to see it used more routinely [3,14,15,16].

A few participants seeking genetic counseling were requesting guidance on which PGx results were worth sharing with family members or physicians. Awareness that PGx test results may have implications for family members has been noted by others [2,20], but participants without prior PGx exposure may confuse PGx testing for disease risk testing [16]. Haga et al. [14] also reported that about two-thirds of participants reported sharing their PGx test results with family members.

Among those who chose to share their PGx results with a physician, a few mentioned that their doctor did not know what to do with the information. In a survey study of patients who had had prior PGx testing conducted by Haga et al. [3], outcomes of PGx result sharing with either pharmacists (25% of participants) or physicians (61% of participants) were captured. About half who shared with a pharmacist felt the pharmacist responded positively and was helpful, while the other half reported either a negative response (no time to review) or no understanding of the result. For those who shared with doctors, 32% reported a positive or helpful response, 29% reported the doctor did not understand the result, and 14% were unsure of the doctor’s response to their PGx result. Other studies capturing participant sharing of PGx test results with healthcare providers have also found that participants report that they are more likely to share results with their prescribing physician rather than a pharmacist [14,15,16]. The expectation among patients for prescribing physicians to be able to respond to PGx test results will have important implications for the broader implementation of PGx testing. A 2016 survey of pharmacy and medical students conducted by Yau and Haque [21] found that over 90% of pharmacy students had a course on PGx, while only 57% of medical students reported the same experience.

A small minority of PGx genetic counseling requests were from participants seeking guidance outside of the scope of genetic counseling practice (7 inquiries, 9%). These requests were regarding whether a dose change may be needed for a currently prescribed medication or whether a change to a different drug may be indicated by their PGx results. Study genetic counselors referred these participants to their prescribing physician or pharmacist to discuss any potential management changes related to their PGx results. As others have observed patients reporting discontinuing or changing medications following PGx testing without the advice of a physician [2], study participants were reminded both in the report text and by genetic counselors to not make any changes to medications without first discussing their results with their physician.

### 4.1. Study Limitations

This study has several limitations. The CPMC cohort is predominantly Caucasian, with relatively high education and income, and therefore not representative of the general public, but likely more representative of current consumers of PGx tests. This study included two methods of delivering genetic counseling—by telephone and e-mail—which could have influenced the type and number of questions asked. The analysis was conducted on written email exchanges between participants and genetic counselors when possible but, in part, relied on session notes taken by genetic counselors. While every effort was made to accurately capture participant questions expressed in telephone genetic counseling sessions, these notes do not capture the interactions verbatim. Ideally, transcripts of phone sessions would have been collected. This study only examines the informational needs of individuals who received preemptive PGx testing via participation in a research study; the needs of patients receiving clinically indicated PGx testing may be different. This study also only captured the questions of the participants who contacted us for genetic counseling and did not capture questions that participants asked other healthcare providers. Report design and content may have also influenced participant understanding of PGx test results. The PGx reports were designed by the CPMC study team, which consisted of several PhD-level genomic scientists and two genetic counselors. Iterative edits were made prior to releasing results to study participants based on feedback received from non-scientific administrative staff who viewed draft reports.

### 4.2. Practice Implications

Most participant questions in this study were able to be addressed by the study genetic counselors with only 7 inquiries (9%) seeking guidance on dosing or drug selection and therefore falling outside of genetic counselor scope of practice. These results lend further support for the partnership between genetic counselors and pharmacists proposed by Mills and Haga [22] to help clinicians in the multi-disciplinary team-based delivery of pharmacogenomics. Pharmacists can utilize their expertise in pharmacokinetics and pharmacodynamics to facilitate the appropriate application of PGx test results to medication selection and dosing, while genetic counselors who are well trained in genetics, risk communication, and patient education can facilitate pre-test discussion of risks, benefits and limitations of testing and post-test discussion of any familial implications, or incidental genomic findings impacting health. Both pharmacists and genetic counselors can lend their unique expertise to assist clinicians with the appropriate use and interpretation of PGx testing.

The data gathered in this study may also provide genetic counselors and other healthcare providers with insight into how to design test result reports and educational materials to better facilitate patient understanding of PGx test results. Use of lay terminology whenever possible, explicitly stating what medications the results apply to and what medications the results do not apply to, and clearly communicating the limitations of PGx testing for predicting drug dosing and response would likely reduce confusion. PGx reports and educational materials should also include more direction on which types of questions physicians, pharmacists, and genetic counselors can each address.

### 4.3. Research Recommendations

Further research should be performed to better understand the PGx informational needs of patients of more diverse racial, ethnic, educational, and socioeconomic status. More investigation is also needed to determine the informational needs of patients undergoing clinically-indicated PGx genetic testing.

Given that an estimated 97% of the population is expected to have an actionable PGx test result [23], further multi-disciplinary work to create scalable tools like online portals or chatbots to deliver PGx results and targeted education will help facilitate the broader clinical implementation of pharmacogenomics.

## 5. Conclusions

Our work illustrates a range of questions raised by study participants receiving PGx test results, most of which were addressed by a genetic counselor with few requiring input from prescribing providers or pharmacists. Genetic counselors may have a role to play in educating physicians and pharmacists on how to effectively communicate with patients about PGx as these are the providers that patients will seek out to manage and explain PGx test results. These results may also lend further support to a role for genetic counselors in a team-based approach to optimal PGx result delivery.

## Figures and Tables

**Table 1:** Participant demographics.

	*n* (5021)	% | Range (SD)
Male	2142	43%
Female	2879	57%
		
Some high school	10	0%
High school graduate	255	5%
Vocational/trade school	28	1%
Some college	678	14%
Associate’s degree	557	11%
Bachelor’s degree	1335	27%
Graduate degree	2152	43%
Do not want to answer	6	0%
		
<USD 25,000	77	2%
USD 25,000–49,999	267	5%
USD 50,000–74,999	357	7%
USD 80,000–99,999	339	7%
equal or >USD 100,000	913	18%
Do not want to answer	3068	61%
		
Mean Age	47	18–94 (15)
		
Caucasian	4395	88%
African American	235	5%
Native American	9	0%
Asian	154	3%
Hawaiian/Pacific Islander	9	0%
Mixed Race	160	3%
Do not want to answer	59	1%

**Table 2:** Genetic counseling (GC) participant demographics.

	All Participants Requesting GC		Participants Requesting PGx GC		Participants Requesting Non-PGx GC	
	*n* (569)	% | Range (SD)	*n* (70)	% | Range (SD)	*n* (499)	% | Range (SD)
Male	201	35%	22	31%	179	36%
Female	368	65%	48	69%	320	64%
						
Some high school	3	1%	0	0%	3	1%
High school graduate	27	5%	9	13%	18	4%
Vocational/trade school	1	0%	0	0%	1	0%
Some college	67	12%	6	9%	61	12%
Associate’s degree	54	9%	7	10%	47	9%
Bachelor’s degree	147	26%	18	26%	129	26%
Graduate degree	268	47%	30	43%	238	48%
Do not want to answer	2	0%	0	0%	2	0%
						
<USD 25,000	25	4%	2	3%	23	5%
USD 25,000–49,999	72	13%	10	14%	62	12%
USD 50,000–74,999	92	16%	7	10%	85	17%
USD 80,000–99,999	111	20%	17	24%	94	19%
equal or > USD 100,000	262	46%	34	49%	228	46%
Do not want to answer	7	1%	0	0%	7	1%
						
Mean age	56	23–91 (13)	58	27–86 (12)	55	23–91 (13)
						
Caucasian	517	91%	64	91%	453	91%
African American	22	4%	1	1%	21	4%
Native American	3	1%	0	0%	3	1%
Asian	6	1%	3	4%	3	1%
Hawaiian/Pacific Islander	0	0%	0	0%	0	0%
Mixed Race	16	3%	2	3%	14	3%
Do not want to answer	5	1%	0	0%	5	1%

**Table 3:** Logistic regression results.

	Eta	Standard Error	*z*-Value	*p*-Value
Intercept	−4.504	0.824	−5.467	0.000
Number of Viewed Reports	0.047	0.020	2.341	0.019
				
Intercept	−6.296	1.980	−3.179	0.001
Number of PGx Report Medications	1.150	0.425	2.706	0.007

## Animal Studies

No non-human animal studies were carried out by the authors for this article.
